# Pulmonary Valve Infective Endocarditis in an Adult Patient with Severe Congenital Pulmonary Stenosis and Ostium Secundum Atrial Septal Defect

**DOI:** 10.4061/2010/798956

**Published:** 2010-12-26

**Authors:** Juan Lacalzada, Cristina Enjuanes, Maria Manuela Izquierdo, Antonio Barragán Acea, Alejandro De La Rosa, Ignacio Laynez

**Affiliations:** Department of Cardiology, University Hospital of the Canary Islands, La Laguna, 38320 Tenerife, Spain

## Abstract

A hypertensive 76-year-old man with severe pulmonary valve stenosis (PVS) and recent initiation of haemodialysis was referred with fever, chills, and asthenia. One month prior, he had been admitted with similar symptoms. Transthoracic echocardiography (TTE) had shown a PVS and no valve vegetations were observed. Following discharge, he was readmitted with fever and blood cultures positive for *Staphylococcus haemolyticus*. A new TTE revealed two pulmonary valve vegetations and a previously undetected ostium secundum-type atrial septal defect (ASD), confirmed by transesophageal echocardiography. The clinical course was uneventful with intravenous antibiotic treatment and the patient was safely discharged. This is a case of pulmonary valve infective endocarditis (IE). The incidence of right-sided IE is on the rise due to the increased number of patients using central venous lines, pacing, haemodialysis and other intravascular devices. Pulmonary valve IE is extremely rare, especially in structurally normal hearts. The case reported here, presents a combination of predisposing factors, such as severe congenital PVS, the presence of a central venous catheter, and haemodialysis. The fact that it was an older patient with severe congenital PVS and associated with a previously undiagnosed ASD, is also an unusual feature of this case, making it even more interesting.

## 1. Case Report

A hypertensive 76-year-old man with severe pulmonary valve stenosis (PVS) and recent initiation of haemodialysis for chronic kidney disease was referred to our hospital with fever, chills, and asthenia. 

**Figure 1:**
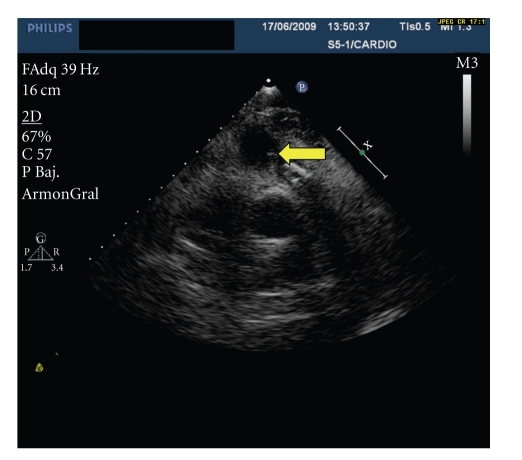
Transthoracic echocardiography parasternal section showing a thickened and calcified pulmonary valve with adhered vegetations.

**Figure 2:**
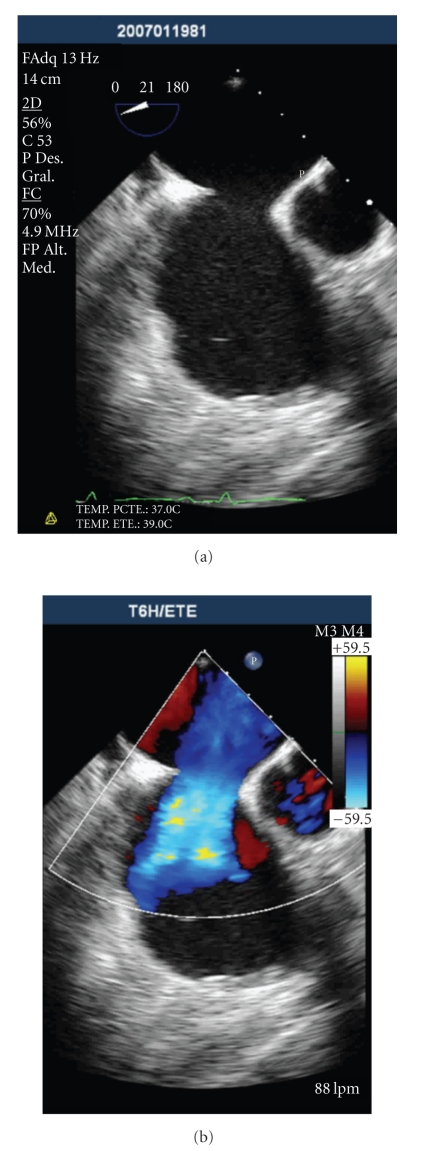
Transesophageal echocardiography showing interatrial communication with severe (a)–(b) shunt.

One month prior, he had been admitted to another hospital with similar symptoms. He was found to have bacteraemia with serial blood cultures positive for *Staphylococcus haemolyticus*, appropriate antibiotic therapy was initiated, and a previously placed right internal yugular hemodialysis catheter was removed. Although transesophageal echocardiography (TEE) was not available in that facility, transthoracic echocardiography (TTE) examination had shown a thickened and calcified pulmonary valve with a reduced opening. Continuous wave Doppler measured a peak systolic gradient of 75 mmHg and mean systolic gradient of 42 mmHg across the pulmonary valve. No valve vegetations were observed. The patient was discharged in good condition 15 days later. Two weeks following discharge, he was readmitted with fever, blood cultures were positive for* Staphylococcus haemolyticus,* and the patient was subsequently referred to our institution. Physical examination on admission was unremarkable, however ultrasonographic examination by TTE revealed two pulmonary valve vegetations (Figure 1). Also noted, were dilated right heart cavities with abnormal interventricular septal motion and normal right ventricular systolic function. A significant left-to-right flow across a previously undetected ostium secundum-type atrial septal defect (ASD) was shown by colour flow Doppler imaging. This findings were confirmed by TEE (Figure 2). 

Blood cultures drawn on admission to our hospital remained positive for *Staphylococcus epidermidis*. He received appropriate intravenous antibiotic treatment for six weeks, and haemodialysis continued throughout an arteriovenous fistula. The clinical course was uneventful and the patient was safely discharged after this time. At present, the patient has refused any intervention on his heart disease, but has remained asymptomatic and without further recurrences.

## 2. Discussion

Right-sided infective endocarditis (RSIE) is a rare condition that constitutes only 10% of cases of IE [1]. It is usually associated with intravenous illicit drug use (IVDU) or central catheter use, with *Staphylococcus aureus* being the most common infectious agent in all cases [2]. 

The incidence of RSIE is on the rise due to the increased number of patients using central venous lines, as well as pacing and other intravascular devices [3]. This incidence is also high among renal patients receiving haemodialysis throughout a long-standing central catheter. The ever increasing number of patients on dialysis requiring central catheters makes RSIE a frequent complication with greater morbidity and mortality than the general population [4]. 

Most cases of RSIE involve the tricuspid valve. By contrast, pulmonary valve endocarditis is extremely rare, especially in structurally normal hearts. Structural cardiac abnormalities such as PVS and ASD were present in our patient and serve as additional risk factors. PVS appears as a rare congenital heart disease in adults, and it is even rarer in association with ASD [5]. 

Another remarkable aspect of this case is the presence of a significant left-to-right shunt through the ASD in a patient with a longstanding severe PVS, due to anticipated changes in right ventricular compliance a smaller shunt would be expected. However, we face the case of an elderly, hypertensive patient with chronic renal failure and associated left ventricular hypertrophy. All of these factors could increase the afterload and lead to a reduced left ventricular compliance. This impaired left compliance could explain the left-to-right shunt severity.

The case of pulmonary valve IE reported here, presents a combination of predisposing factors for this entity, such as severe congenital PVS, the presence of a central venous catheter, and haemodialysis. The fact that it was an older patient with severe congenital PVS and associated with a previously undiagnosed ASD, is also an unusual feature of this case, making it even more interesting. Interatrial communication has been reported in some cases of RSIE; however, these involved normal native pulmonary valves [6]. Anatomically normal, bicuspid, or even unicuspid pulmonary valves have been found in another series of nine autopsies of patients with isolated pulmonary valves IE, but they were not associated with neither PVS nor ASD [7]. To our knowledge, the association of both structural heart diseases leading to pulmonary valve IE had not been previously reported. 

RSIE usually presents without the classic signs and symptoms of IE, and therefore, the diagnosis is often more challenging [8]. Other possible septic sources as well as other septic clinical complications such as clinical or radiological septic pulmonary embolism, which may be associated with RSIE, were reasonably ruled out in this case. The diagnosis of pulmonary valve IE was made based on the presence of two major and two minor modified Duke criteria [3]. The challenge in diagnosing pulmonary valve IE, as compared to other valvular locations, which may lead to a low clinical suspicion and cause it to be overlooked. The initial TTE was performed in another institution, so we cannot determine whether the valvular vegetation was simply overlooked or was not present. Importantly, in this particular case our initial clinical suspicion and a detailed echocardiography imaging study allowed us to establish the definitive diagnosis and led us to initiate a successful treatment of IE and obtain a favourable outcome.

In conclusion, the pulmonary valve should be properly assessed in TTE and TEE examinations of all patients with clinical suspicion of IE, especially in the presence of prolonged fever associated with pulmonary infectious processes, IVDU, central catheters, or haemodialysis, and particularly if they also have predisposing heart disease. The diagnosis of pulmonary valve IE should no longer be missed in cases similar to this one. Failure of this diagnosis could be the main reason for such low frequency of RSIE.
